# The apoB100/apoAI ratio is independently associated with the severity of coronary heart disease: a cross sectional study in patients undergoing coronary angiography

**DOI:** 10.1186/s12944-015-0155-6

**Published:** 2015-11-18

**Authors:** Yongyan Song, Yang Yang, Jingxiao Zhang, Yanmei Wang, Wenfeng He, Xiaoming Zhang, Jie Zhu, Zhan Lu

**Affiliations:** Department of Medical Biochemistry, School of Preclinical Medicine, North Sichuan Medical College, Nanchong, 637000 P. R. China; School of Clinical Medicine, North Sichuan Medical College, Nanchong, 637000 P. R. China; Department of Cardiology, Affiliated Hospital of North Sichuan Medical College, Nanchong, 637000, Sichuan Province P. R. China; College of Medicine, Yaan Vocational and Technical College, Yaan, 625000 P. R. China; Sichuan Key Laboratory of Medical Imaging, and Department of Radiology, Affiliated Hospital of North Sichuan Medical College, Nanchong, 637000, Sichuan Province China

**Keywords:** Lipoprotein ratio, ApoB100/apoAI, Coronary heart disease, Severity, Gensini score

## Abstract

**Background:**

Lipoprotein ratios have been shown to be associated with the occurrence of coronary heart disease (CHD), but little is known about their relationships with the severity of CHD.

**Methods:**

A total of 792 angiographically defined CHD patients were enrolled following their admission. Patients were stratified into three groups based on the tertile of the Gensini scores (≤33^rd^ percentile, 33^rd^ to 66^th^ percentile and ≥66^th^ percentile) or the number of stenotic coronary branches (single-branch stenosis, double-branch stenosis and multi-branch stenosis). Demographic and biochemical data were collected and lipoprotein ratios were calculated. Logistic regression and path analysis were employed to examine the relationships between the lipoprotein ratios and the severity of CHD.

**Results:**

The ratios of low-density lipoprotein cholesterol (LDL-C)/high-density lipoprotein cholesterol (HDL-C) and apolipoprotein B100 (apoB100)/apolipoprotein AI (apoAI) increased with the tertile of the Gensini scores (*P* < 0.05 for both). The ratios of triglyceride (TG)/HDL-C, total cholesterol (TC)/HDL-C, LDL-C/HDL-C and apoB100/apoAI increased with the number of stenotic coronary branches (*P* < 0.05 for all). The univariate logistic regression showed that the ratios of TC/HDL-C, LDL-C/HDL-C and apoB100/apoAI were positively associated with both the tertile of the Gensini scores and the number of stenotic vessels (*P* < 0.05 for all), and the ratio of TG/HDL-C was positively associated with the number of stenotic vessels (*P* < 0.05). In multivariate logistic analysis, only the ratio of apoB100/apoAI was independently and positively associated with the tertile of the Gensini scores (OR = 2.93, 95 % CI = 1.17-7.34, *P* = 0.022) and the number of stenotic vessels (OR = 3.14, 95 % CI = 1.01-6.47, *P* = 0.048) after adjusting for the possible confounding variables. The apoB100/apoAI ratio was also shown to be a direct mediator between the risk factors including age, BMI, HDL-C, LDL-C, apoB100 and apoAI and the severity of CHD by path analysis.

**Conclusion:**

Our data indicate that the apoB100/apoAI ratio could be a useful predictor for evaluating the severity of coronary stenosis in CHD patients.

## Background

Coronary heart disease (CHD) is the major cause of death in developed countries and some developing countries like China [1]. CHD is recognized as a multifactorial disease, and dyslipidemia is closely associated with the progression of coronary atherosclerosis. Yusuf S and colleagues [2] demonstrated that dyslipidemia could account for around 50 % of the population-attributable risk for CHD. Reliable indexes for assessing the CHD risk and the extent of coronary stenosis are pivotal to manage and prevent this critical disease. The roles of plasma low-density lipoprotein cholesterol (LDL-C), total cholesterol (TC), high-density lipoprotein cholesterol (HDL-C) and triglycerides (TG) in the development of CHD have been well established. Currently, increases in LDL-C, TC, TG and/or decreases in HDL-C are widely used to evaluate the risk of CHD in clinical practice. According to the 2013 ACC/AHA blood cholesterol guidelines [3] and the Adult Treatment Panel III (ATP III) Guidelines [4] of the United States, LDL-C is considered as the major cause of CHD and used as the primary target for therapy, and the other lipid parameters were used as the secondary or supplementary targets.

Although there were growing arguments that the lipoprotein ratios, which have atherogenic components (TG, TC and LDL-C) in the numerator and antiatherogenic components (HDL-C) in the denominator, could be better predictors for the occurrence [5–7] and development of CHD [8–12] than the individual lipid parameters, the roles of the lipoprotein ratios in the pathogenesis of CHD are still obscure. One point to support these arguments is that the lipoprotein ratios can comprehensively reflect the balance between the atherogenic and antiatherogenic potentials in one individual. An increase in TG, TC or LDL-C concentrations is an atherogenic lipid marker, whereas reduced HDL-C concentration is an antiatherogenic trait which may also be correlated with numerous other cardiovascular risk factors, including the components of the metabolic syndrome. In an angiography-based study, Lippi U et al. [8] were the first to report that TC/HDL-C and LDL-C/HDL-C are statistically related to the severity of CHD. Their findings were later proved by another study [9] from Zhejiang University, China, in which the researchers demonstrated that the ratios of LDL-C/HDL-C and TC/HDL-C were significantly and positively correlated with the coronary artery lesions. A recent cross-sectional association study [10] in a Lebanese population further revealed that the TC/HDL-C ratio ≥4 significantly predicts ≥50 % stenosis in all vessels. The ratio of TG/HDL-C was also found to be associated with the severity of CHD in two studies from Ruijin Hospital, Shanghai Jiaotong University, China [11] and Insituto do Coração, Universidade de São Paulo, Brazil [12], respectively.

However, none of these studies on outcomes have evaluated the association of the apolipoprotein B100 (apoB100)/apolipoprotein AI (apoAI) ratio with the severity of CHD. In theory, the apoB100/apoAI ratio should be an even better predictor for evaluating the severity of CHD than the HDL-C related ratios. Each particle of the atherogenic lipoproteins [low-density lipoprotein, very low-density lipoprotein, intermediary density lipoprotein and lipoprotein (a)] carries one apoB100 molecule, so the concentrations of plasma apoB100 can reflect the total number of atherogenic particles. On the other hand, apoAI accounts for about 70 % of the total apolipoproteins in high-density lipoprotein; the plasma content of apoAI represents the total of antiatherogenic particles. Therefore, the apoB100/apoAI ratio might be a superior marker for CHD development in that it can comprehensively reflect the balance between the atherogenic particles and antiatherogenic particles. Several studies [13–16] have suggested that the apoB100/apoAI ratio is better than the HDL-C related ratios in predicting the occurrence of CHD. In a prospective apolipoprotein-related mortality risk (AMORIS) study [13], the researchers reported that the apoB100/apoAI ratio was strongly related to increased coronary risk, and was superior to any of the HDL-C related ratios in predicting CHD in a large sample of 69,030 men and 57,168 women above 40 years of age. Liem AH et al. [14] demonstrated that the apoB100/apoAI, but not the LDL-C/HDL-C ratio, is positively associated with cardiovascular events in statin-treated patients with known CHD.

We previously demonstrated that the lipoprotein ratios are superior to the individual lipid parameters as predictors for CHD occurrence, and the apoB100/apoAI ratio is the best one to predict CHD [7]. In the current study, we further investigated the associations between the ratios of TG/HDL-C, TC/HDL-C, LDL-C/HDL-C and apoB100/apoAI and the severity of CHD. We hypothesized that higher levels of TG/HDL-C, TC/HDL-C, LDL-C/HDL-C, and especially apoB100/apoAI are associated with more severe CHD.

## Results

### Clinical characteristics of the study population

The CHD patients were divided into three groups according to the tertile of the Gensini scores: group 1: ≤ 33^rd^ percentile (scores ≤ 11.5); group 2: 33^rd^ to 66^th^ percentile (scores 12–32); group 3: ≥66^th^ percentile (scores ≥ 33). The anthropometric and biochemical characteristics of the three groups are shown in Table 1. The mean age, and the prevalence of male gender and smoking increased with the tertile of the Gensini scores (*P <* 0.05 for all). The ratios of LDL-C/HDL-C and apoB/apoAI increased, whereas HDL-C and apoAI decreased with the tertile of the Gensini scores (*P <* 0.05 for all). There were no significant differences in weight, BMI, prevalence of hypertension and diabetes, TG, TC, LDL-C, apoB100, TG/HDL-C and TC/HDL-C across the groups.

**Table 1:** Clinical characteristics of the CHD patients according to the tertile of the Gensini scores

Variables	Group 1	Group 2	Group 3	*P* value
	≤33^rd^ percentile (*n* = 265)	33^rd^ to 66^th^ percentile (*n* = 261)	≥66^th^ percentile (*n* = 266)	
Non-lipid variables				
Age, years	62.06 ± 9.98	62.68 ± 10.61	64.95 ± 9.67	0.003
Male gender, n (%)	171 (64.5)	172 (65.9)	204 (76.7)	0.004
Weight, kg	64.95 ± 9.29	63.39 ± 9.36	64.67 ± 9.63	0.225
BMI, kg/m^2^	24.56 ± 2.84	24.15 ± 3.03	24.16 ± 3.04	0.399
Smokers, n (%)	95 (35.8)	120 (46.0)	133 (50.0)	0.003
Hypertension, n (%)	119 (44.9)	141 (54.0)	138 (51.9)	0.091
Diabetes, n (%)	35 (13.2)	34 (13.0)	51 (19.2)	0.080
Individual lipid variables				
TG, mmol/L	1.53 ± 0.90	1.49 ± 1.09	1.66 ± 1.14	0.156
TC, mmol/L	4.16 ± 0.94	4.22 ± 1.01	4.23 ± 1.20	0.728
HDL-C, mmol/L	1.01 ± 0.29	1.02 ± 0.34	0.93 ± 0.29	0.001
LDL-C, mmol/L	2.37 ± 0.75	2.44 ± 0.81	2.49 ± 0.94	0.263
ApoAI, g/L	1.03 ± 0.19	1.02 ± 0.21	0.95 ± 0.21	<0.001
ApoB100, g/L	0.76 ± 0.24	0.77 ± 0.25	0.80 ± 0.28	0.204
Lipoprotein ratios				
TG/HDL-C	1.83 ± 2.06	2.09 ± 4.41	2.16 ± 2.53	0.466
TC/HDL-C	4.51 ± 1.92	4.73 ± 3.58	5.03 ± 3.18	0.079
LDL-C/HDL-C	2.57 ± 1.11	2.70 ± 1.75	2.96 ± 2.01	0.024
ApoB/apoAI	0.76 ± 0.27	0.78 ± 0.29	0.88 ± 0.43	<0.001

The differences across the tertile of the Gensini scores were calculated by Chi-square test for categorical variables, and one-way ANOVA analysis for continuous variables

*BMI* body mass index, *TG* triglycerides, *TC* total cholesterol, *HDL-C* high-density lipoprotein cholesterol, *LDL-C* low-density lipoprotein cholesterol, *apoAI* apolipoprotein AI, *apoB100* apolipoprotein B100

The anthropometric and biochemical characteristics of the CHD patients with different number of stenotic vessels are shown in Table 2. The mean age, and the prevalence of male gender, cigarette smoking, hypertension and diabetes increased with the number of stenotic vessels (*P <* 0.05 for all). TG, TC, LDL-C, apoB100, the ratios of TG/HDL-C, TC/HDL-C, LDL-C/HDL-C and apoB/apoAI increased, and HDL-C and apoAI decreased with the number of stenotic vessels (*P <* 0.05 for all). There were no significant differences in weight and BMI across the groups.

**Table 2:** Clinical characteristics of the CHD patients with different number of stenotic vessels

Variables	Single-branch stenosis (*n* = 327)	Double-branch stenosis (*n* = 212)	Multi-branch stenosis (*n* = 253)	*P* value
Non-lipid variables				
Age, years	61.93 ± 10.25	63.04 ± 9.56	65.12 ± 10.25	0.001
Male gender, n (%)	197 (60.2)	158 (74.5)	193 (76.3)	<0.001
Weight, kg	64.26 ± 9.62	64.32 ± 8.71	64.43 ± 9.81	0.985
BMI, kg/m^2^	24.43 ± 2.80	24.40 ± 3.15	24.31 ± 3.13	0.923
Smokers, n (%)	119 (36.4)	103 (48.6)	123 (48.6)	0.003
Hypertension, n (%)	155 (47.4)	97 (45.8)	142 (56.1)	0.045
Diabetes, n (%)	37 (11.3)	32 (15.1)	51 (20.2)	0.013
Individual lipid variables				
TG, mmol/L	1.44 ± 0.80	1.63 ± 1.08	1.67 ± 1.30	0.049
TC, mmol/L	4.07 ± 0.87	4.32 ± 1.10	4.30 ± 1.22	0.028
HDL-C, mmol/L	1.03 ± 0.308	0.99 ± 0.33	0.94 ± 0.30	0.003
LDL-C, mmol/L	2.31 ± 0.73	2.50 ± 0.83	2.56 ± 0.97	0.004
ApoAI, g/L	1.03 ± 0.20	1.00 ± 0.20	0.96 ± 0.22	0.001
ApoB100, g/L	0.74 ± 0.24	0.79 ± 0.25	0.80 ± 0.29	0.009
Lipoprotein ratios				
TG/HDL-C	1.64 ± 1.36	2.20 ± 2.70	2.43 ± 5.05	0.003
TC/HDL-C	4.26 ± 1.50	5.10 ± 4.01	5.12 ± 3.33	<0.001
LDL-C/HDL-C	2.44 ± 1.03	2.87 ± 1.83	3.05 ± 3.05	<0.001
ApoB/apoAI	0.74 ± 0.26	0.81 ± 0.28	0.88 ± 0.45	<0.001

The differences across the groups with different number of stenotic vessels were calculated by Chi-square test for categorical variables, and one-way ANOVA analysis for continuous variables

*BMI* body mass index, *TG* triglycerides, *TC* total cholesterol, *HDL-C* high-density lipoprotein cholesterol, *LDL-C* low-density lipoprotein cholesterol, *apoAI* apolipoprotein AI, *apoB100* apolipoprotein B100

### Univariate logistic regression of the lipoprotein ratios and the severity of CHD

Univariate logistic regression analyses showed that the ratio of TG/HDL-C was significantly associated with the number of stenotic vessels (*P <* 0.05), but not with the tertile of the Gensini scores. The ratios of TC/HDL-C, LDL-C/HDL-C and apoB100/apoAI were significantly associated with both the tertile of the Gensini scores and the number of stenotic vessels (*P <* 0.05 for all) (Table 3).

**Table 3:** Results of the univariate ordinal logistic regression of the lipoprotein ratios and the tertile of the Gensini scores or the number of stenotic vessels

Variables	The tertile of the Gensini scores			The number of stenotic vessels		
	OR	95 % CI	*P*	OR	95 % CI	*P*
TG/HDL-C	1.02	0.98–1.07	0.341	1.05	1.01–1.09	0.021
TC/HDL-C	1.05	1.00–1.10	0.034	1.09	1.04–1.14	<0.001
LDL-C/HDL-C	1.12	1.02–1.23	0.017	1.20	1.10–1.31	<0.001
ApoB100/apoAI	2.30	1.51–3.51	<0.001	3.09	2.07–4.61	<0.001

*OR* odds ratio, *95 % CI* 95 % confidence interval, *TG/HDL-C* triglyceride/high-density lipoprotein cholesterol, *TC/HDL-C* total cholesterol/high-density lipoprotein cholesterol, *LDL-C/HDL-C* low-density lipoprotein cholesterol/high-density lipoprotein cholesterol, *apoB100/apoAI* apolipoprotein AI/apolipoprotein B100

### Multivariate logistic regression of the lipoprotein ratios and the severity of CHD

As shown in Table 4, the ratios of TC/HDL-C and LDL-C/HDL-C were significantly associated with both the tertile of the Gensini scores and the number of stenotic vessels, and TG/HDL-C was significantly associated with the number of stenotic vessels after adjusting for the non-lipid cardiovascular risk factors including age, gender, weight, BMI, smoking, alcohol consumption, diabetes mellitus and hypertension (model 1). However, all the associations between the ratios of TG/HDL-C, TC/HDL-C and LDL-C/HDL-C and the severity of CHD became insignificant after adjusting for the variables in model 1 plus the individual lipid variables including TG, HDL-C, LDL-C, apoB100 and apoAI. TC was excluded during the model-building process due to its significant collinearity with LDL-C. ApoB100/apoAI was independently and significantly associated with the tertile of the Gensini scores and the number of stenotic vessels after adjusting for the variables in model 2 plus the lipoprotein ratios including TG/HDL-C and LDL-C/HDL-C (Table 4). TC/HDL-C was excluded from the model as it is highly correlated with LDL-C/HDL-C.

**Table 4:** Results of multivariate ordinal logistic regression of the lipoprotein ratios and the tertile of the Gensini scores or the number of stenotic vessels

Variables	The tertile of the Gensini scores			The number of stenotic vessels		
	OR	95 % CI	*P*	OR	95 % CI	*P*
TG/HDL-C						
Model 1	1.02	0.98–1.08	0.339	1.05	1.00–1.10	0.033
Model 2	0.98	0.91–1.05	0.576	1.00	0.93–1.07	0.983
Model 3 ^a^	0.98	0.91–1.05	0.536	1.04	0.90–1.21	0.600
LDL-C/HDL-C						
Model 1	1.16	1.02–1.31	0.019	1.39	1.22–1.58	<0.001
Model 2	0.99	0.827–1.19	0.909	1.01	0.83–1.22	0.960
Model 3 ^b^	1.13	0.77–1.66	0.533	0.87	0.57–1.31	0.499
TC/HDL-C						
Model 1	1.07	1.01–1.14	0.030	1.13	1.06–1.21	<0.001
Model 2	0.97	0.89–1.72	0.485	0.99	0.91–1.08	0.888
Model 3 ^b^	0.87	0.65–1.16	0.334	0.81	0.59–1.10	0.176
ApoB100/apoAI						
Model 1	2.73	1.60–4.66	<0.001	4.79	2.80–8.20	<0.001
Model 2	3.18	1.30–7.76	0.011	2.55	1.04–6.28	0.041
Model 3 ^c^	2.93	1.17–7.34	0.022	3.14	1.01–6.47	0.048

Model 1, adjusted for the non-lipid CHD risk factors including age, gender, weight, BMI, smoking, alcohol consumption, diabetes mellitus and hypertension; Model 2, adjusted for the variables in Model 1 plus the individual lipid variables including TG, HDL-C, LDL-C, apoB100 and apoAI; Model 3 ^a^, adjusted for the variables in Model 2 plus the lipoprotein ratios including LDL-C/HDL-C and apoB100/apoAI; Model 3 ^b^, adjusted for the variables in Model 2 plus the lipoprotein ratios including TG/HDL-C and apoB100/apoAI; Model 3 ^c^, adjusted for the variables in Model 2 plus the lipoprotein ratios including TG/HDL-C and LDL-C/HDL-C

*OR* odds ratio, *95 % CI* 95 % confidence interval, *TG/HDL-C* triglyceride/high-density lipoprotein cholesterol, *TC/HDL-C* total cholesterol/high-density lipoprotein cholesterol, *LDL-C/HDL-C* low-density lipoprotein cholesterol/high-density lipoprotein cholesterol, *apoB100/apoAI* apolipoprotein AI/apolipoprotein B100

### Path analysis of the lipoprotein ratios and the severity of CHD

**Fig. 1:**
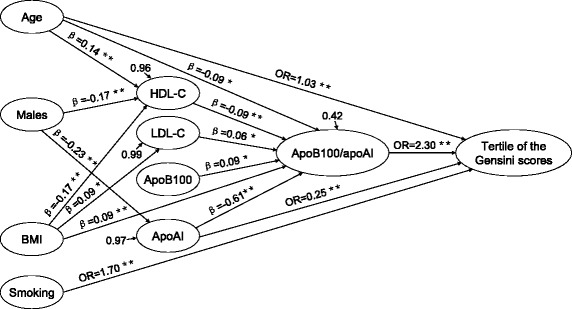
Significant regression paths among dependent (the tertile of the Gensini scores) and independent variables. ^*^ *P* ≤ 0.05, ^**^ *P* ≤ 0.01, standardized regression coefficients (β) or odds ratios (OR) represented as one-way arrows

**Fig. 2:**
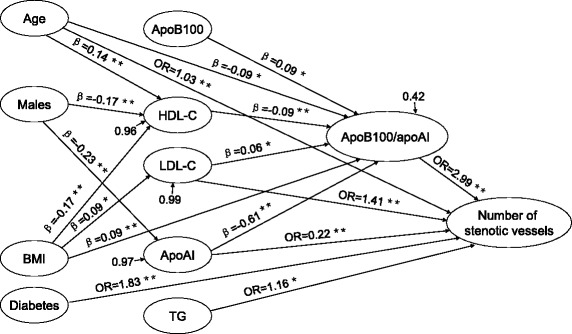
Significant regression paths among dependent (the number of stenotic vessels) and independent variables. ^*^ *P* ≤ 0.05, ^**^ *P* ≤ 0.01, standardized regression coefficients (β) or odds ratios (OR) represented as one-way arrows

Path analysis indicated that age, smoking, diabetes, TG, LDL-C, apoAI and apoB100/apoAI had direct effects on the severity of CHD. Among them, age, smoking, apoAI and apoB100/apoAI had direct effect on the tertile of the Gensini scores (Fig. 1), and age, diabetes, TG, LDL-C, apoAI and apoB100/apoAI had direct effect on the number of stenotic branches (Fig. 2). The variables including age, male gender, BMI, HDL-C, LDL-C, apoB100 and apoAI also had indirect effects on the severity of CHD mediated by the apoB100/apoAI ratio. There were 11 paths (age → apoB100/apoAI → the tertile of the Gensini scores or the number of stenotic vessels, BMI → apoB100/apoAI → the tertile of the Gensini scores or the number of stenotic vessels, HDL-C → apoB100/apoAI → the tertile of the Gensini scores or the number of stenotic vessels, age → HDL-C → apoB100/apoAI → the tertile of the Gensini scores or the number of stenotic vessels, male gender → HDL-C → apoB100/apoAI → the tertile of the Gensini scores or the number of stenotic vessels, BMI → HDL-C → apoB100/apoAI → the tertile of the Gensini scores or the number of stenotic vessels, LDL-C → apoB100/apoAI → the tertile of the Gensini scores or the number of stenotic vessels, BMI → LDL-C → apoB100/apoAI → the tertile of the Gensini scores or the number of stenotic vessels, apoAI → apoB100/apoAI → the tertile of the Gensini scores or the number of stenotic vessels, male gender → apoAI → apoB100/apoAI → the tertile of the Gensini scores or the number of stenotic vessels, and apoB100 → apoB100/apoAI → the tertile of the Gensini scores or the number of stenotic vessels) through which apoB100/apoAI mediated the indirect effects of the CHD risk factors on the severity of CHD.

## Discussion

We have previously established a predictive role of lipoprotein ratios for the occurrence of CHD [7]. We found that the lipoprotein ratios were superior to individual lipid parameters as predictors for CHD, and apoB100/apoAI was the best one to predict the CHD risk. In the present study, the associations between the lipoprotein ratios and the severity of CHD were further investigated and the results showed that the ratio of apoB100/apoAI, but not the HDL-C related ratios (i.e. TG/HDL-C, TC/HDL-C and LDL-C/HDL-C), was significantly and independently associated with the severity of CHD. The statistical power calculations revealed that there was ≥ 87 % power to detect a significant association between the ratio of apoB100/apoAI and the severity of CHD (α = 0.05) with an effect size of ORs ≥ 2.30 in univariate and multivariate analyses. The design of our study is unique in that both the Gensini scores and the number of stenotic coronary branches were employed to evaluate the severity of CHD in one study.

To the best of our knowledge, this study is the first one indicating that the apoB100/apoAI ratio is a strong predictor for the severity of CHD in Chinese Han people. The Han people are the largest ethnic group in East Asia, constituting approximately 92 % of the population of Mainland China, 98 % of the population of Taiwan, and 74 % of the population of Singapore. Previous studies have shown that high apoB100/apoAI ratio is independently correlated with the presence of angiographic CHD [7, 13, 17, 18]. In the current study, we further revealed that this ratio index is independently associated with the severity of CHD. The apoB100/apoAI ratio increased orderly with the severity of stenosis expressed by the tertile of the Gensini scores (Table 1), but also with the number of stenotic coronary branches (Table 2). In univariate logistic regression, the apoB100/apoAI ratio showed a strong relationship with the tertile of the Gensini scores and the number of stenotic vessels (Table 3), and this association was not appreciably altered after adjusting for age, gender, weight, BMI, smoking, alcohol consumption, diabetes mellitus, hypertension, TG, LDL-C, HDL-C, TG/HDL-C and LDL-C/HDL-C in the following multivariate logistic regression (Table 4). Taken together, these results indicate that the apoB100/apoAI ratio could be a strong predictor for the severity of CHD.

In path analysis, we found that some conventional cardiovascular risk factors such as male gender, BMI, HDL-C, LDL-C, apoB100 and apoAI had indirect effects on the severity of CHD, and apoB100/apoAI acted as a key mediator in this process. Age and apoAI can directly affect the severity of CHD, and they also had indirect effects on the severity of CHD through the mediation of apoB100/apoAI. In addition, the results of the path analysis showed that smoking and diabetes had significant effects on the severity of CHD independently of the ratio of apoB100/apoAI and other lipid parameters.

It is theoretically reasonable that the ratio of apoB100/apoAI is strongly associated with the severity of CHD. Each particle of the atherogenic lipoproteins, such as low-density lipoprotein, very low-density lipoprotein, intermediate-density lipoprotein and lipoprotein(a), carries one apoB100 molecule, so the concentrations of plasma apoB100 can reflect the total of proatherogenic potentials. On the other hand, apoAI is the major apolipoprotein component of antiatherogenic lipoprotein, i.e. high-density lipoprotein, the serum content of apoAI represents the total of antiatherogenic potentials. Thus, the apoB100/apoAI ratio could comprehensively reflect the balance between the atherogenic and antiatherogenic potentials in one individual. The higher is the apoB100/apoAI ratio, the more are the atherogenic potentials, or the less are the antiatherogenic potentials, or both.

The ratio of TG/HDL-C was found to be associated with the number of stenotic vessels, but not the tertile of the Gensini scores by univariate logistic regression (Table 3). In multivariate logistic regression, TG/HDL-C was significantly associated with the number of stenotic vessels after adjusting for the non-lipid cardiovascular risk factors including age, gender, weight, BMI, smoking, alcohol consumption, diabetes mellitus and hypertension in model 1. However, it became insignificant when the individual lipid parameters (i.e. TG, LDL-C, HDL-C, apoB100 and apoAI) were added to the model (model 2), which indicates that the predictive power of TG/HDL-C to the number of stenotic vessels can be replaced by the individual lipid parameters. Our findings are consistent with the results of a hospital-based study [9], in which Yang D et al. demonstrated that the TG/HDL-C ratio was not associated with the Gensini scores in Chinese CHD patients. However, in another case–control study which was also involved in Chinese CHD patients, Yunke Z et al. [11] reported that the TG/HDL-C ratio can predict the severity of CHD and the incident of new-onset heart failure among in-hospital CHD patients. In Brazilians, da Luz PL et al. [12] also demonstrated that the TG/HDL-C ratio was associated with the severity of CHD. The association between the TG/HDL-C ratio and the severity of CHD needs to be further studied.

The ratios of TC/HDL-C and LDL-C/HDL-C were associated with both the tertile of the Gensini scores and the number of stenotic vessels in univariate logistic regression analyses (Table 3). In the following multivariate logistic regression, the significant associations persisted when controlled for the non-lipid variables in model 1. However, the associations were completely abolished for both TC/HDL-C and LDL-C/HDL-C when individual lipid variables were added in model 2. The associations between the ratios of TC/HDL-C and LDL-C/HDL-C and the severity of CHD could be mediated by the individual lipid parameters. Contrary to our findings, the significant associations between the ratios of TC/HDL-C and LDL-C/HDL-C and the severity of CHD were reported by other investigators. Yang D et al. [9] reported a significant and positive correlation between the ratios of TC/HDL-C and LDL/HDL-C and the Gensini scores in Chinese patients. Lippi U et al. [8] demonstrated that the TC/HDL-C and LDL/HDL-C ratios, but not the plasma levels of TG, TC, HDL-C and LDL-C are statistically related to the mild severity of CHD. In a recent cross-sectional association study [10], Platt DE et al. reported that the TC/HDL-C ratio is a strong biological marker for CHD occurrence and severity, and the TC/HDL-C ratio ≥ 4 significantly predicts ≥ 50 % stenosis in all vessels. However, apoB100 and apoAI were not enrolled into the regression models in all the above-mentioned studies [8–10], so it is not clear that the associations between the TC/HDL-C and LDL/HDL-C ratios and the severity of CHD were independent of apoB100 and apoAI.

Our study has several limitations. First, the present study was not designed to examine all the risk factors associated with the severity of CHD. Rather, the specific goal was to examine whether the lipoprotein ratios were associated with the severity of CHD. Second, our study mainly focused on the CHD patients whose lesions of coronary artery were 50 % or more in at least one major branch according to the diagnostic criteria. Those with mild coronary stenosis (less than 50 %) were not enrolled in this study although the lipoprotein ratios might also be associated with mild stenosis.

## Conclusions

In conclusion, the results of our study suggest that the ratio of apoB100/apoAI could be a powerful predictor for the severity of CHD.

## Patients and methods

### CHD patients

Details of this study have been published previously [7]. Briefly, a total of 792 consecutive unrelated atherosclerotic CHD patients who underwent coronary angiography at the Affiliated Hospital of North Sichuan Medical College (Nanchong, China) were enrolled in the study between May 2011 and April 2015. Patients taking lipid lowering drugs or the drugs that might affect the glucose or lipid metabolism were excluded from the study. In order to enlarge the sample size, the patients who took the drugs which were thought not to affect plasma lipid levels were still enrolled in the study. Patients with renal or hepatic dysfunction, significant valvular disease, myocarditis, and malignant disease were excluded from the study. All the subjects were Chinese Han people. The tenets of the Declaration of Helsinki were adhered to in all procedures in the study. The study protocol was approved by the ethics committee of North Sichuan Medical College. Written consent was provided by all the participants or their guardians prior to their participation in the study.

### Coronary angiography

The Gensini scoring system and the number of stenotic coronary branches were used to assess the severity of CHD. Patients were stratified into three groups based on the tertile of the Gensini scores or the number of stenotic coronary branches. Coronary angiograms were evaluated by experienced cardiologists who were not aware of the patients’ biochemical status. Standard coronary angiography with at least two views of the right coronary artery and four views of the left coronary system were performed using the Judkins technique by Allura Xper FD20 (Philips Medical Systems Nederland B.V. Netherlands). Atherosclerotic CHD was diagnosed in patients who had angiographic evidence of stenosis greater than 50 % in at least one major coronary artery. Those with normal coronary arteries or minimal stenosis (less than 50 %) in any of the major coronary arteries were excluded from the study.

### Diagnostic criteria

Body mass index (BMI) was calculated by dividing weight by height squared (kg/m^2^). Smoking was defined as regular cigarette smoking. Hypertension was defined as the measurement of systolic/diastolic blood pressure higher than 140/90 mmHg or active use of antihypertensive drugs. Diabetes mellitus was defined as the fasting glucose levels above 126 mg/dL or active use of antidiabetic drugs or insulin.

### Biochemical measurement

Fasting blood samples were taken on the first morning of the in-hospital day when lipid-lowering drugs were not yet used. Samples were immediately transported to the Department of Clinical Laboratory of the Affiliated Hospital of North Sichuan Medical College for measurement of plasma lipids. TG, TC, LDL-C and HDL-C were measured directly by enzymatic methods. ApoB100 and apoAI were measured by immunoturbidimetric assays. All the measurements were carried out using an automatic clinical chemistry analyzer (Beckman Coulter AU5800, USA). Lipoprotein ratios were calculated.

### Statistical analysis

Data are presented as mean ± standard deviation (SD) unless otherwise stated. Continuous variables were tested for normality; otherwise, log transformation was applied. All statistical analyses were carried out by using SPSS version 13.0 (SPSS Inc., Chicago, IL, USA). The differences among the three groups categorized by the tertile of the Gensini scores or the number of stenotic vessels were calculated by Chi-square test for categorical variables, and one-way ANOVA analysis for continuous variables. Univariate and multivariate ordinal logistic regression analyses were employed to determine the associations between the lipoprotein ratios and the severity of atherosclerotic CHD. The results of logistic regression analysis were expressed as the odds ratio (OR) with 95 % confidence interval (95 % CI). In the multivariate regression, collinearity among the independent variables was diagnosed by examining variance inflation factor (VIF) and tolerance. Path analysis was used to examine the causal relationships among the non-lipid cardiovascular risk factors, individual lipid variables, lipoprotein ratios and the severity of CHD. Power calculations were performed using the PASS 14 program based on Hsieh’s method [19]. All *P*-values are two-tailed and differences were considered significant if *P* ≤ 0.05.

## Abbreviations

CHD: Coronary heart disease
BMI: Body mass index
TG: Triglycerides
TC: Total cholesterol
HDL-C: High-density lipoprotein cholesterol
LDL-C: Low-density lipoprotein cholesterol
ApoB100: Apolipoprotein B100
ApoAI: Apolipoprotein AI
